# QiDongNing induces lung cancer cell apoptosis via triggering P53/DRP1‐mediated mitochondrial fission

**DOI:** 10.1111/jcmm.18353

**Published:** 2024-04-29

**Authors:** Rongzhen Ding, Yichao Wang, Ling Xu, Shuliu Sang, Guanjin Wu, Wenxiao Yang, Yilu Zhang, Chengyan Wang, Ao Qi, Haiping Xie, Yi Liu, Aiguo Dai, Lijing Jiao

**Affiliations:** ^1^ Department of Oncology, Yueyang Hospital of Integrated Traditional Chinese and Western Medicine Shanghai University of Traditional Chinese Medicine Shanghai China; ^2^ Institutional Key Laboratory of Vascular Biology and Translational Medicine in Hunan Province Hunan University of Chinese Medicine Changsha China; ^3^ Department of Respiratory Diseases, Medical School Hunan University of Chinese Medicine Changsha China; ^4^ Institute of Translational Cancer Research for Integrated Chinese and Western Medicine, Yueyang Hospital of Integrated Traditional Chinese and Western Medicine Shanghai University of Traditional Chinese Medicine Shanghai China

**Keywords:** apoptosis, lung cancer, mitochondrial fission, p53, QiDongNing

## Abstract

Non‐small‐cell lung cancer (NSCLC) is a major cause of worldwide cancer death, posing a challenge for effective treatment. Our previous findings showed that Chinese herbal medicine (CHM) QiDongNing (QDN) could upregulate the expression of p53 and trigger cell apoptosis in NSCLC. Here, our objective was to investigate the mechanisms of QDN‐induced apoptosis enhancement. We chose A549 and NCI‐H460 cells for validation in vitro, and LLC cells were applied to form a subcutaneous transplantation tumour model for validation in more depth. Our findings indicated that QDN inhibited multiple biological behaviours, including cell proliferation, cloning, migration, invasion and induction of apoptosis. We further discovered that QDN increased the pro‐apoptotic BAX while inhibiting the anti‐apoptotic Bcl2. QDN therapy led to a decline in adenosine triphosphate (ATP) and a rise in reactive oxygen species (ROS). Furthermore, QDN elevated the levels of the tumour suppressor p53 and the mitochondrial division factor DRP1 and FIS1, and decreased the mitochondrial fusion molecules MFN1, MFN2, and OPA1. The results were further verified by rescue experiments, the p53 inhibitor Pifithrin‐α and the mitochondrial division inhibitor Mdivi1 partially inhibited QDN‐induced apoptosis and mitochondrial dysfunction, whereas overexpression of p53 rather increased the efficacy of the therapy. Additionally, QDN inhibited tumour growth with acceptable safety in vivo. In conclusion, QDN induced apoptosis via triggering p53/DRP1‐mediated mitochondrial fission in NSCLC cells.

## 1 INTRODUCTION

Non‐small‐cell lung cancer (NSCLC) is a major cause of worldwide cancer death, posing a challenge for effective treatment.^1^ NSCLC is the predominant histotype in lung cancer, making up 85% of the cases. The adaptive growth of tumour cells, exhibiting infinite proliferation, is fundamental for poor prognosis. Apoptosis plays a crucial role in the growth, multiplication and regeneration of malignant cells, and the ability to evade apoptosis seems to be a critical process in the development of tumours and the advancement of cancer.^2^ Even though radiotherapy, molecular targeted therapy and immunotherapy have been proven to induce cell apoptosis, tumour cells still have many adaptive responses to the continuously changing tumour microenvironment, turning on anti‐apoptotic self‐defence mechanisms and triggering drug resistance. Therefore, new treatment modalities are urgently needed.

Mitochondria, the energy factories of cells, are closely associated with cancer cell survival and progression.^3^ Evidence suggests that mitochondrial metabolic function is among the most important factors for tumour cell apoptosis, and targeting mitochondrial functional metabolism is expected to be a new direction for cancer therapy.^4^ Mitochondrial dynamics is a constant process that regulates mitochondrial morphology, function and number through division and fusion in a dynamic equilibrium.^5^ Multiple GTPases, such as Dynamin‐Related Protein 1 (DRP1), Mitofusin 1/2 (MFN1/2) and Optic Atrophy 1 (OPA1), regulate the division and fusion of mitochondria.^6^ The majority of irregularities in mitochondrial dynamics result from the disruption of the mitochondrial membrane, inhibition of the respiratory chain, reduction in enzyme activity and damage of mitochondrial DNA. These factors lead to impaired energy metabolism, loss of control over cell differentiation and growth and disruptions in the cell cycle.^7^, ^8^ The combination of fused mitochondrial structure is associated with increased ATP generation and reduced ROS levels, whereas fragmented mitochondrial structure results in mitochondrial uncoupling, decreased mitochondrial membrane potential and impaired ATP production, ultimately leading to apoptosis.^9^

Deletion of the p53 gene causes mitochondrial oxidative phosphorylation and glycolysis, thus impairing metabolic reprogramming and mitochondrial metabolic function in malignant cells.^10^ Activating pro‐apoptotic factors and inducing mitochondrial outer membrane permeabilization are important functions of p53. These functions are achieved through transcriptional regulation and direct interactions, leading to the initiation of apoptotic pathways both internally and externally.^11^ Various stimuli in the mitochondrial apoptosis pathway recruit p53 to the nucleus, where it participates in the transcription of multiple apoptotic factors, including BAX, PUMA, NOXA and Apaf1.^12^ In addition, p53 regulates the post‐translational gene expression of anti‐apoptotic proteins and also functions as a repressor of anti‐apoptotic factors including Bcl2, Bcl‐xL and Mcl‐1.^13^, ^14^

Chinese herbal medicine (CHM) is efficacious in reducing toxicity and delaying drug resistance in current lung cancer treatments and can also activate various apoptosis modulators and initiate various apoptosis‐related transduction pathways. QiDongNing (QDN) was formulated in our previous study by optimizing the formula of the anti‐lung cancer Chinese medicine ‘Jinfu Kang’.^15^ The various components of QDN have been found to possess a broad spectrum of anti‐cancer effects, including the ability to induce apoptosis and inhibit the proliferation, migration, and invasion of NSCLC cells.^16^ Our preceding findings have acknowledged that QDN can induce apoptosis in NSCLC cells by increasing the tumour suppressor p53 expression. The mechanism is possibly connected with the upregulation of apoptosis‐related proteins Fas, Bax, Caspase3, Caspase8 and the downregulation of Bcl‐2 expression. The study was designed to investigate the impacts and underlying process of QDN‐induced apoptosis in NSCLC cells regarding mitochondrial dynamics, organelle morphology, cellular function and metabolism, and to elucidate the involvement of p53/DRP1 in initiating mitochondrial division.

## 2 MATERIALS AND METHODS

### 2.1 Detection of main components in QDN

QDN is composed of *Astragalus, Radix Ophiopogonis, Paris polyphylla, Glossy Privet Fruit and Fiveleaf Gynostemma*. The preparation of QDN followed the previously described method, which involved using a weight ratio of 3:1:2:1:1 for the herbs.^17^ We used UHPLC (Nexera UHPLC LC‐30A) to collect the group of square mass spectrometry data. The constituents of QDN were examined and identified by conducting searches on the databases of the China National Knowledge Infrastructure (CNKI) and PubMed. The Waters HSS T3 column was utilized with electrospray ionization (ESI) being applied in both positive and negative ion modes. In the experiment, the ion source parameters were as follows: For positive ion mode, the ion spray voltage (IS) was adjusted to 5500 V and for negative ion mode to −4500 V. The temperature was consistently maintained at 450°C. Furthermore, the pressure for atomization (GAS1) and the pressure for heater gas (GAS2) were both adjusted to 55 psi, whereas the pressure for curtain gas (CUR) was set to 35 psi. The voltage for de‐clustered (DP) was adjusted to 80 V while the collision energy (CE) was set at 10 eV. The survey scans for time‐of‐flight (TOF)‐MS were performed in the m/z range of 60–1000.

### 2.2 Reagents and antibodies

The p53 inhibitor Pifithrin‐α (PFT‐α) was obtained from Beyotime Biotechnology and was dissolved in DMSO. Mdivi1, a compound that inhibits mitochondrial fission, was obtained from MedChemExpress. It was then added to the cell medium at a concentration of 10 μM for 12 h. The mitochondrial antioxidant Mitoquinone (MitoQ) was purchased from MedChemExpress (Shanghai, China) and dissolved in DMSO to a concentration of 50 nM. Necrostatin‐1 was acquired from MedChemExpress and dissolved in DMSO to a concentration of 40 μM. Anti‐DRP1 (8570), Anti‐OPA1 (80471), Anti‐MFN1 (14739), Anti‐BAX (5023), Anti‐BCL2 (15071) and Anti‐GAPDH (5174) were provided by Cell Signaling Technology. Anti‐p53 (sc‐126) was supplied by Santa Cruz Biotechnology Inc. Anti‐rabbit IgG (HRP‐conjugated) (ab6721) and anti‐mouse IgG (HRP‐conjugated) (ab6789) were obtained from Abcam.

### 2.3 Cell culture

The NSCLC cell lines utilized in this work were sourced from the National Collection of Authenticated Cell Cultures. The Lewis lung carcinoma (LLC) was acquired from the Cell Bank of the Chinese Academy of Sciences. AusGeneX provided RPMI medium supplemented with 10% foetal bovine serum (v/v) for culturing the NSCLC cells. The cells were cultured in an incubator with a humidity level of 5% CO_2_.

### 2.4 Cell counting Kit‐8 assay

The Cell Counting Kit‐8 (CCK8) assay (Sangon Biotech) was used to measure cell proliferation as described previously.^15^ NSCLC cells were planted in transparent 96‐well plates and placed in an incubator for 24 h. Subsequently, the cells were cultured with various concentrations of QDN (0, 62.5, 125, 250, 500, 1000 μg/mL) for 48 h after affixing the plate. CCK8 reagent was added to each well and incubated for 2 h in a CO_2_ light‐protected incubator. The optical density (OD) absorbance in 450 nm wavelength was detected by a microplate reader (Multiskan GO, Thermo Fisher Scientific). Inhibition ratio (%) = (1‐ODsample/ODcontrol) × 100%. The IC_50_ value was measured by GraphPad Prism 8.0.

### 2.5 Colony formation assay

A colony formation experiment was applied to investigate tumour cells' clonogenic potential. Briefly, cells were added in 6‐well plates at 500 cells/well and incubated with QDN for 48 h. Medium with 30% FBS was added every 3–4 days to the culture plate for 14 days. A549 and NCI‐H460 were revealed with crystal violet after 4% PFA fixation (Sangon). Image J was then used to count the number of visible colonies.

### 2.6 Scratch wound assay

Cells were seeded in 12‐well plates when they reached 80% confluence. Subsequently, scratch wounds were created by scraping with a 10 μL pipette tip. The scratches were photographed after 24 h incubation of QDN at IC_50_ concentrations.

### 2.7 Invasion assay

A transwell chamber assay was conducted to measure the invasive property. A layer of Matrigel (Thermo Fisher Scientific) weighing 1 μg was applied to the top of the transwell chamber membrane (8 μm, Corning) before use. In the upper chamber, the FBS‐free RPMI medium was used to seed a total of 100,000 cells, whereas the lower chamber was replenished with 500 μL of complete medium. QDN was added to the upper chambers and incubated for 48 h. Following the incubation period, a soft cotton‐tipped swab was employed to eliminate non‐intrusive cells from the upper chamber. Meanwhile, the cells that successfully penetrated the membrane and reached the lower chamber were treated with PFA and 0.05% crystal violet (Sangon). Invaded cells that crossed the chamber were captured and counted.

### 2.8 Apoptosis analysis

NSCLC cells were cultivated in QDN for 48 h at an IC50 concentration before being harvested. The apoptosis detection kit (Sangon) was employed following the experimental procedure. Flow cytometry (BD Biosciences) was utilized to evaluate cell apoptosis, and the acquired data were analysed using FlowJo v10 software.

### 2.9 ROS measurement

The ROS Assay Kit (Beyotime) was utilized to evaluate intracellular ROS levels. Cells were administrated with QDN at 1/2 IC_50_ and IC_50_ concentrations for 48 h. To quantify the levels of reactive oxygen species (ROS), the harvested cells were treated with a fluorescent probe called DCFH‐DA. Subsequently, the fluorescence intensity was measured using a multimode microplate reader (TECAN, Infinite M200).

### 2.10 ATP measurement

The ATP content was determined by employing an ATP assay kit (Beyotime) as per the instructions provided with the kit. QDN was administered to cells at concentrations of 1/2 IC_50_ and IC_50_ for 48 hours. To measure ATP levels, whole‐cell lysates were obtained in 96‐well plates with a solid black surface. The cellular ATP content was then evaluated using a multimode microplate reader (TECAN, Infinite M200).

### 2.11 Mitochondrial membrane potential (△Ψm) assay

The JC‐1 assay kit (Beyotime) was employed to evaluate the alteration in the mitochondrial membrane potential (MMP, △Ψm) by measuring the change in the JC‐1 levels. JC‐1 selectively enters mitochondria, exhibiting different fluorescence properties in accordance with changes in MMP. With elevated MMP, JC‐1 polymerizes and exhibits a crimson luminescence. While with lower MMP, it exists as a monomer and fluoresces green. The cells were subjected to QDN concentrations for a period of 48 hours. Cells were then examined and visualized with an inverted Leica AF6000 epifluorescence microscope (Leica).

### 2.12 Quantification of mitochondrial morphology

The Mito‐Tracker Green (Beyotime) was utilized for mitochondria‐specific fluorescent staining of living cells. Images were acquired using an inverted Leica AF6000 epifluorescence microscope (Leica). The ImageJ was exploited to quantify mitochondrial morphological changes.^18^

### 2.13 Western blotting

RIPA lysis buffer was employed to prepare the protein extracts, which were then determined by a BCA kit. The proteins were separated by electrophoresis on a 10% SDS‐PAGE gel and subsequently transferred onto a PVDF (polyvinylidene fluoride) membrane. The membranes were treated with suitable primary antibodies and incubated overnight at 4°C, followed by blocking with 5% non‐fat milk. After incubating with HRP‐conjugated secondary antibodies (Invitrogen; Thermo Fisher Scientific Inc.), immunoreactive bands were observed using the ECL Western blot kit.

### 2.14 Lentiviral transfection

The lentivirus plasmid (Han Heng Biological Science & Technology Co. Ltd.) was used to infect A549 and NCI‐H460 cells in order to enhance the expression of p53, following the provided protocol. In short, the cells were cultured with HBLV‐p53‐PURO, along with polybrene at a concentration of 8 ug/ml. Finally, transfected cells were selected by puromycin (1.5 ug/ml).

### 2.15 Tumour growth assays

Six‐week‐old male C57 BL/6 mice (Shanghai Jihui Laboratory Animal Care Co., Ltd.) were grown in an SPF condition. This study was approved and supervised by the animal ethics committee of Yueyang Hospital of Integrated Traditional Chinese and Western Medicine, Shanghai University of Traditional Chinese Medicine. The treatment of animals in all experiments conforms to the ethical standards of experimental animals. On the right side of every mouse, 0.1 mL of PBS containing LLC cells (5 × 10^5^) was injected subcutaneously. After the tumours were grown to a mean size of 50 mm^3^, animals were separated into three groups at random (*n* = 10 per arm). The mice were given intragastric administration with QDN‐L (9.4 g/kg) and QDN‐H (18.8 g/kg), from the next day for 14 consecutive days. Tumour volume was determined by a vernier calliper and was measured utilizing the modified ellipsoid formula: tumour volume (mm^3^) = length×width^2^ × 0.5. At the endpoint, all mice were put to death by cervical dislocation, and the major organs were harvested for HE staining, tumours were taken out for weight, and immunohistochemistry.

### 2.16 TUNEL assay

The TUNEL Immunostaining was conducted with the TUNEL assay kit (Invitrogen; Thermo Fisher Scientific, Inc.).The sections were incubated with TUNEL reagents at a temperature of 37°C for 1 h, and the nucleus was counterstained using DAPI. Pictures were taken by fluorescence microscopy (Zeiss). The apoptotic rate is measured as the number of apoptotic cells divided by the total number of cells and multiplied by 100%.

### 2.17 Statistical analysis

Statistical software GraphPad Prism 8.0 was utilized for conducting data analysis and visualization. The mean standard deviation (SD) represents the outcomes of three distinct experiments. To determine group disparities, one‐way ANOVA and two‐way ANOVA with repeated measures were utilized for experiments. A *p*‐value of 0.05 reached statistical significance.

## 3 RESULTS

### 3.1 Chemical composition identification of QDN

Through UHPLC analysis of QDN samples, a total of 98 non‐polar compounds were characterized, including 37 compounds attributable to *Astragalus*, 31 to *Radix Ophiopogonis*, 16 to *Paris polyphylla*, 18 to *Glossy Privet Fruit* and 31 to *Fiveleaf Gynostemma* (Table S1). These results demonstrated that QDN is rich in saponins, oleic acids and esters, which are probably important factors in the anti‐tumour effects of QDN. The total ion chromatograms are shown in Figure S1.

### 3.2 QDN exerts an anti‐lung cancer effect by suppressing various biological behaviours of NSCLC cells

**FIGURE 1:**
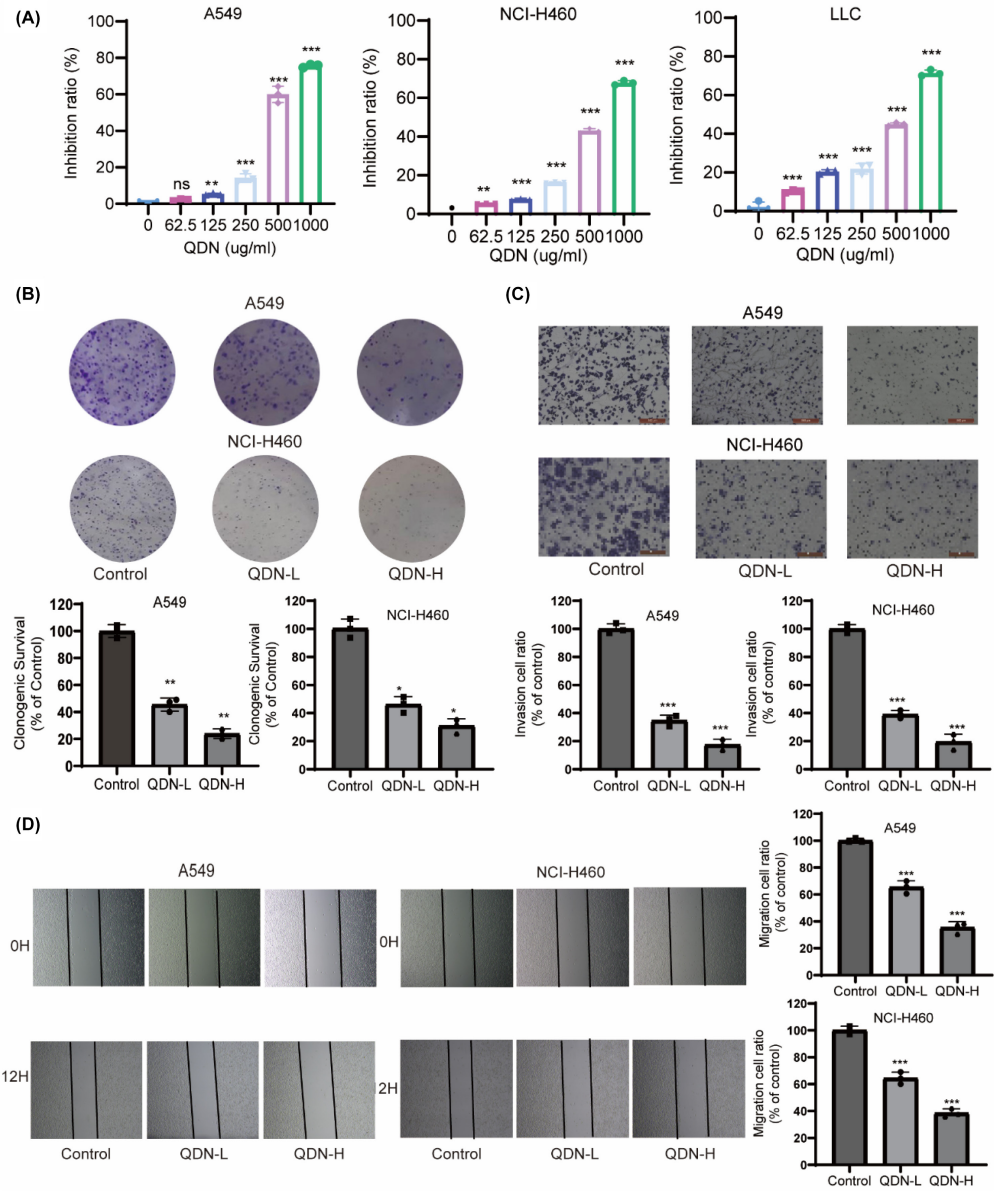
QDN exerts an anti‐lung cancer effect by affecting various biological behaviours of lung cancer cells. (A) Dose–response curves of A549, NCI‐H460 and LLC cell lines for 48 h with varying concentrations of QDN. (B) Clonogenic capacity of A549 and NCI‐H460 cells after 48 h treatment with QDN. (C) Invasive ability of A549 and NCI‐H460 cells treated with QDN for 48 h. (D) The typical wound scratches in A549 and NCI‐H460 cells after treatment with QDN for 24 h. Data are mean ± SD of duplicate experiments (*n* = 3). Parametric variables were calculated using one‐way ANOVA. *p < 0.05, **p < 0.01, ***p < 0.001, ns, no significant difference, compared with control group. QDN‐H, high dose of QDN; QDN‐L, low dose of QDN.

We first carried out the cell proliferation experiment to identify the efficacy of QDN on cancer cell growth, including A549, NCI‐H460 and LLC. As indicated in Figure 1A, cell proliferation was greatly suppressed by QDN in contrast to the control group in a dose‐dependent manner (IC_50_ in Table S2). The following in vitro experiments were performed with QDN‐L (1/2IC_50_) and QDN‐H (IC_50_) in A549 and H460 cells.

Clonogenicity is an important indicator to investigate cell survival and replicative potential.^19^ As illustrated in Figure 1B, QDN‐L and QDN‐H reduced the proportion of A549 cell colonies from 100% to 46.51% and 25.59%, and that of NCI‐H460 cells from 100% to 48.77% and 32.25% respectively, implicating QDN dose‐dependently inhibited the clonogenic ability of NSCLC cells.

To verify QDN's efficacy on cell invasion, the transwell chamber test was employed. The number of invading cells significantly decreased following treatment with QDN in comparison with the control cells (Figure 1C).

To assess the ability of QDN on cell migration, scratch wound assays were applied in NSCLC cells with QDN treatment. Cells incubated with QDN exerted significantly less migration ability in comparison with control cells (Figure 1D). The findings indicate that the anti‐lung cancer effect of QDN is related to the suppression of cell proliferation, cloning, migration and invasion.

### 3.3 QDN induces NSCLC cell apoptosis and modulates mitochondrial dynamics

**FIGURE 2:**
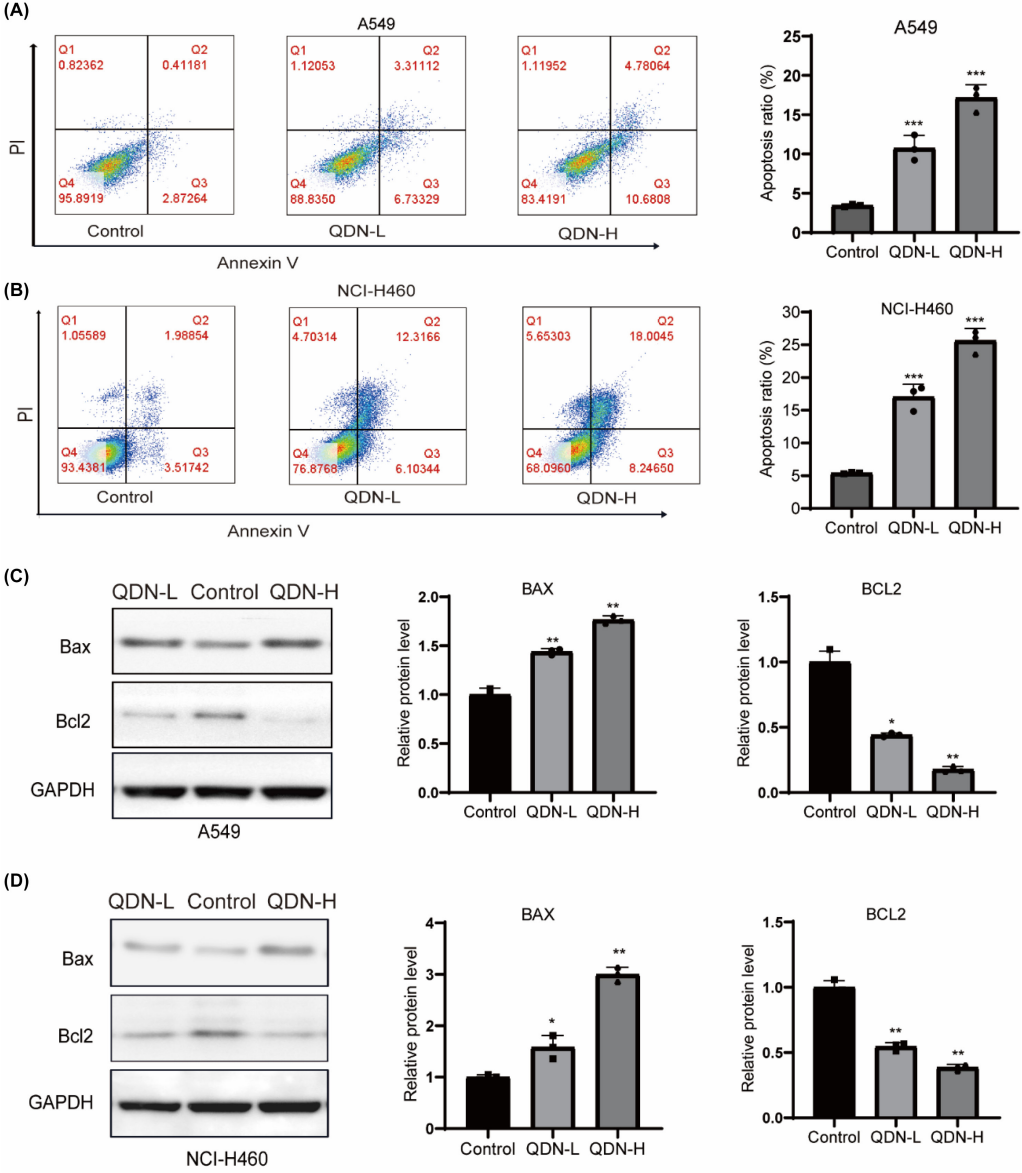
QDN induces apoptosis in NSCLC cells. (A,B) The apoptotic cell proportion of A549 and NCI‐H460 was determined. (C,D) Protein expression of Bax and Bcl2 after QDN treatment for 48 h. Data are mean ± SD of duplicate experiments (*n* = 3). Parametric variables were calculated using one‐way ANOVA. *p < 0.05, **p < 0.01, ***p < 0.001, compared with control group. QDN‐H, high dose of QDN; QDN‐L, low dose of QDN.

To demonstrate whether decreased viability was related to apoptosis induction, AnnexinV/PI staining was performed. As presented in Figure 2A,B, QDN‐L and QDN‐H increased the apoptotic rates of A549 cells to 10.74% and 17.49%, and that of NCI‐H460 cells to 17.71% and 26.63% respectively, compared with the control cells. In line with Annexin V‐FITC/PI double labelling, QDN upregulated Bax but strongly suppressed the level of Bcl2 (Figure 2C,D).

In addition, we verified the effect of QDN on necroptosis in lung cancer cells. The necroptosis inhibitor Necrostatin‐1 (40 μM) were added to the A549 and NCI‐H460 cells 2 h prior to QDN treatment (QDN‐H). We determined cell activity using the CCK8 assay which indicated that the combination of QDN with Necrostatin‐1 failed to reduce cell inhibition. We have also detected RIPK1, a key protein in necroptosis, which further indicated no effect of QDN on necroptosis (Figure S2A–D).

**FIGURE 3:**
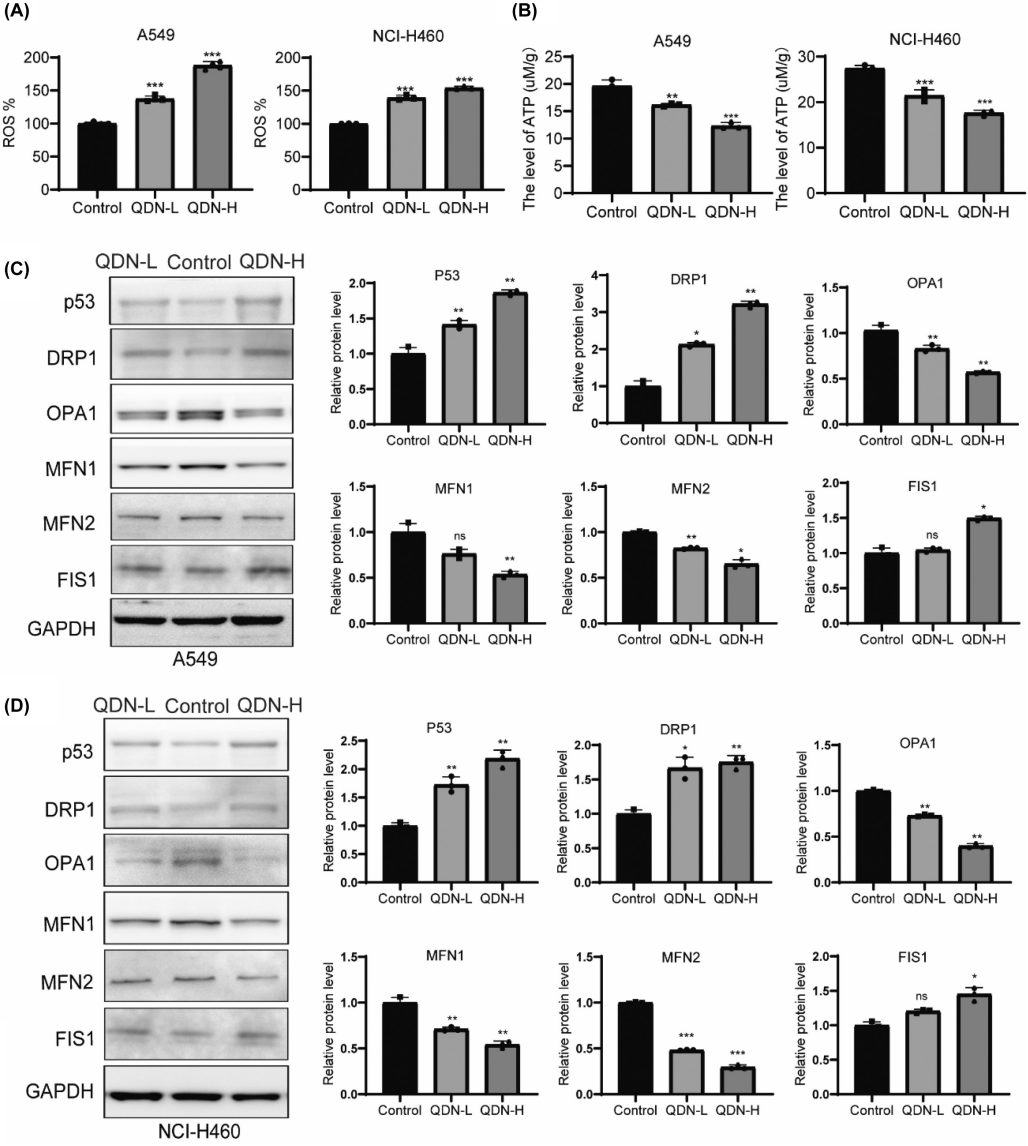
QDN evokes mitochondrial dysfunction and altered dynamics. (A) ROS production rates in A549 and NCI‐H460 cells with QDN treatment for 48 h. (B) ATP levels in A549 and NCI‐H460 cells with QDN treatment for 48 h. (C,D) Protein expression of p53, DRP1, OPA1, MFN1, MFN2 and FIS1 in A549 and NCI‐H460 cells after QDN treatment for 48 h. Data are mean ± SD of duplicate experiments (*n* = 3). Parametric variables were calculated using one‐way ANOVA. *p < 0.05, **p < 0.01, ***p < 0.001, ns, no significant difference, compared with control group. QDN‐H, high dose of QDN; QDN‐L, low dose of QDN.

ROS level was considerably increased, and ATP level was decreased after QDN treatment, in comparison with the control group (Figure 3A,B). Disturbance of homeostasis results in ROS accumulation and ATP reduction, leading to mitochondrial damage, which is crucial in cancer progression.^20^

To analyse the possible mechanism of QDN, the total protein levels of p53 and mitochondrial proteins were detected by western blot. Mitochondrial dynamics are tightly regulated by a variety of factors, including mitochondrial fusion proteins MFN1, MFN2 and OPA1, and mitochondrial fission protein DRP1 and FIS1.^21^, ^22^ As illustrated in Figure 3C,D, QDN increased the level of p53, DRP1and FIS1 and reduced the level of OPA1, MFN1 and MFN2. We also extracted mitochondria from A549 and NCI‐H460 cells. P53 levels in the mitochondrial fraction were assessed using western blotting, revealing no significant changes (Figure S2E,F). Thus, QDN‐induced apoptosis is mainly associated with cytoplasmic p53.

These findings indicated that mitochondrial dynamics are responsible for QDN‐induced apoptosis in lung cancer cells.

### 3.4 P53 is involved in QDN‐induced apoptosis on NSCLC cells

**FIGURE 4:**
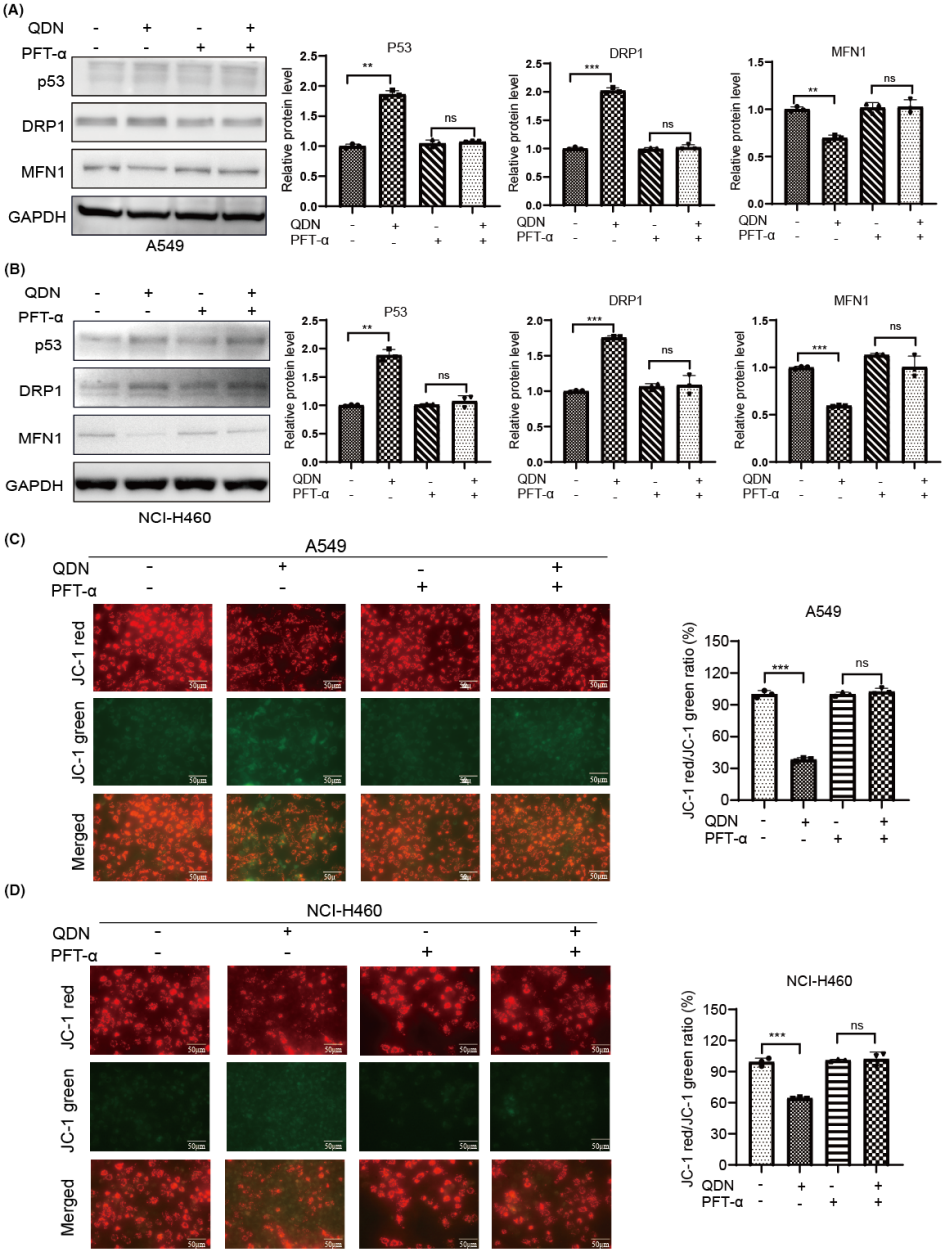
P53 inhibition partially attenuates the effect of QDN on mitochondrial function. (A,B) Protein expression of p53, DRP1 and MFN1 in A549 cells and NCI‐H460 cells after QDN administration for 48 h with or without PFTα pretreatment. (C,D) MMP level after QDN treatment for 48 h pretreated with or without PFT‐α. Data are mean ± SD of duplicate experiments (*n* = 3). Parametric variables were calculated using one‐way ANOVA. *p < 0.05, **p < 0.01, ***p < 0.001, ns, no significant difference. QDN‐H, high dose of QDN; QDN‐L, low dose of QDN.

Given the preliminary evidence of the suppressive effect of QDN on various biological behaviours of lung cancer cells and the apoptosis induction, as well as the upregulation of p53, we assumed that QDN plays a regulatory role in NSCLC through p53 activation. Previous results revealed that QDN significantly exerted anti‐tumour effects in a dose‐dependent manner. Next, we explored the molecular mechanism using the IC_50_ concentration of QDN. As shown in Figure 4A,B, pretreatment with p53 inhibitor PFT‐α (20 μM, 24 h) produced a minimal influence effect on mitochondrial dynamism protein. The downregulation of MFN1 by QDN treatment was counteracted after the PFT‐α combination. Similarly, the efficacy of QDN in increasing DRP1 level was antagonized when QDN was combined with PFT‐α. To further investigate the mechanism of apoptosis induced by QDN, the MMP level was measured by JC‐1 staining. As displayed in Figure 4C,D, an decrease was observed in JC‐1 red/JC‐1 green ratio QDN treatment in lung cancer cells, and the effect on cellular MMP level was counteracted by PFT‐α pretreatment.

**FIGURE 5:**
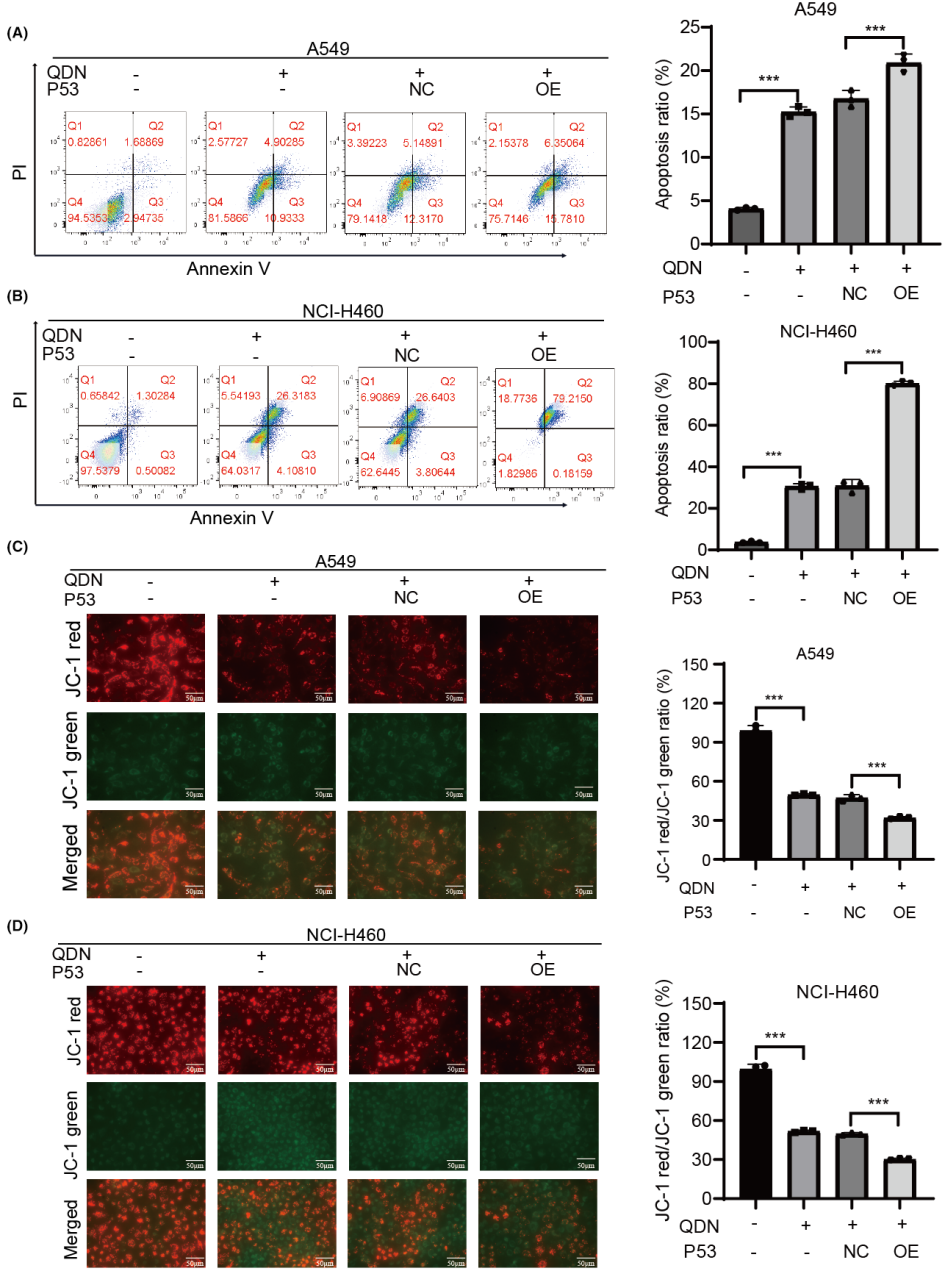
p53 overexpression enhances the efficacy of QDN on cell apoptosis. (A,B) The apoptotic cell proportion of A549 and NCI‐H460 cells overexpressing p53 after QDN treatment for 48 h. (C,D) MMP level in p53 overexpressed A549 and NCI‐H460 cells after QDN treatment for 48 h. NC: p53 NC, OE: p53 overexpression. Data are mean ± SD of duplicate experiments (*n* = 3). Parametric variables were calculated using one‐way ANOVA. *p < 0.05, **p < 0.01, ***p < 0.001, compared with control group. QDN‐H, high dose of QDN; QDN‐L, low dose of QDN.

To confirm the role of p53 in cell apoptosis and mitochondrial dynamics by QDN, the lentiviral plasmid was used to establish p53 overexpressed NSCLC cells. In QDN‐treated cells, p53 overexpressing A549 cells exhibited more apoptotic cells, and this efficacy was more obvious in H460 cells, implying that the ability of QDN to induce apoptosis is significantly elevated by p53 overexpression (Figure 5A,B). Compared with control cells, QDN induced a significant loss of MMP in p53 overexpressing cells (Figure 5C,D). These results further demonstrate that QDN exerts apoptosis induction and mitochondrial dynamics regulation through p53 upregulation.

### 3.5 Mitochondrial fission inhibition partially attenuates the effect of QDN on NSCLC cells

**FIGURE 6:**
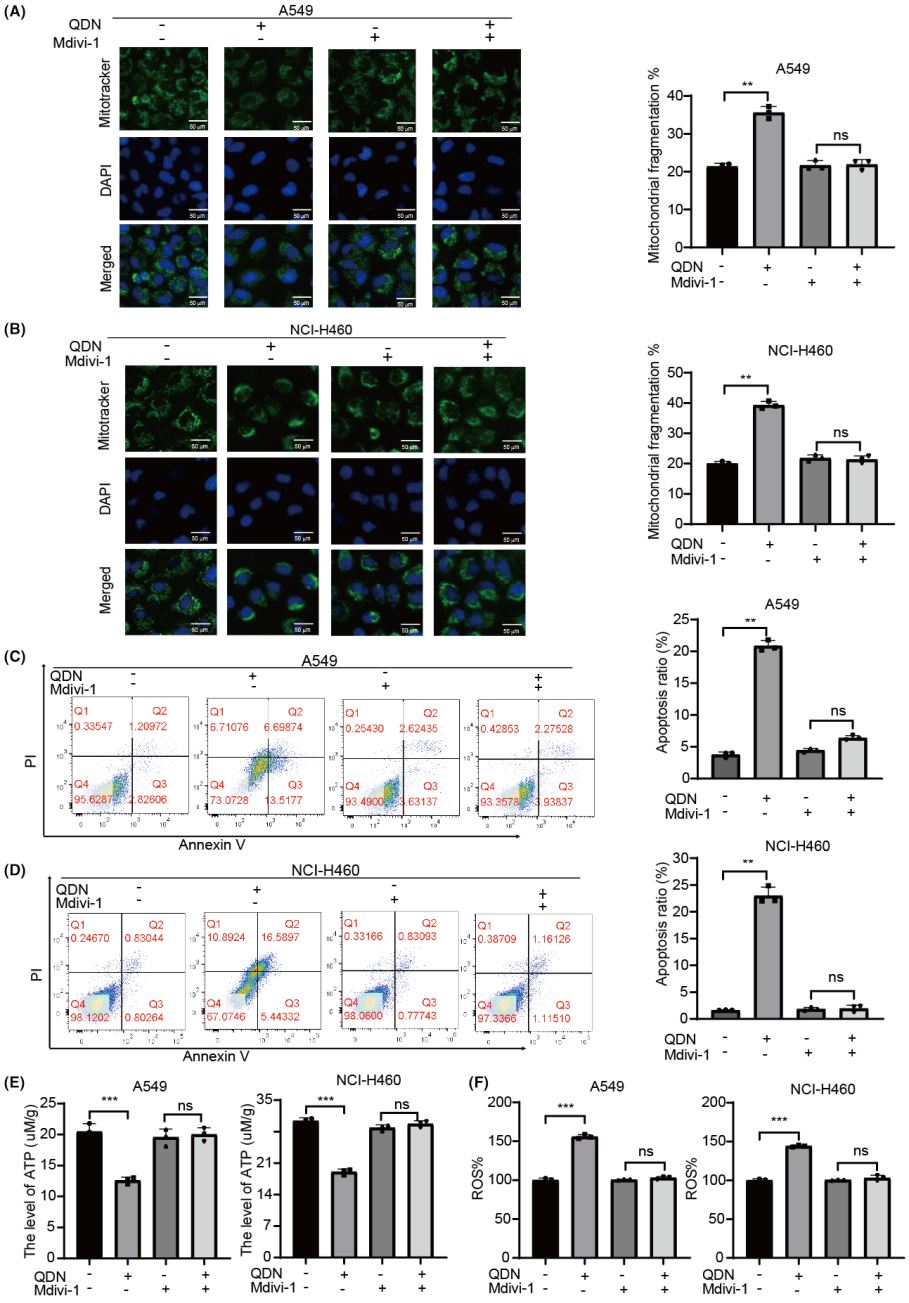
Inhibition of mitochondrial fission partially counteracts the effect of QDN on mitochondrial dynamics. (A,B) The mitochondrial morphology of A549 and NCI‐H460 cells after QDN treatment for 48 h with or without Mdivi‐1 pretreatment. (C,D) The apoptotic cell proportion of A549 and NCI‐H460 cells after QDN treatment for 48 h pretreated with or without Mdivi‐1. (E) ATP levels in A549 and NCI‐H460 cells after QDN treatment for 48 h with or without Mdivi‐1 pretreatment. (F) ROS production rates in A549 and NCI‐H460 cells after QDN treatment for 48 h with or without Mdivi‐1 pretreatment. Data are mean ± SD of duplicate experiments (*n* = 3). Parametric variables were calculated using one‐way ANOVA. *p < 0.05, **p < 0.01, ***p < 0.001, ns, no significant difference. QDN‐H, high dose of QDN; QDN‐L, low dose of QDN.

Although the mitochondria are in a constant state of transition between fission and fusion, excessive mitochondrial fission leads to a decrease in metabolic function and an increase in oxidative stress. To further evaluate the impact of mitochondrial fission in QDN‐induced apoptosis in lung cancer, NSCLC cells were cultured with mitochondrial fission inhibitors Mdivi1 (10 μM, 12 h). As shown in Figure 6A,B, Mdivi1 had little effect on mitochondrial division. Cotreatment with QDN and Mdivi1 significantly reduced circular and punctate mitochondrial fragmentation when compared to QDN alone. Further, the apoptotic induction by QDN was also observed to be counteracted by Mdivi1 (Figure 6C,D). Mdivi1 suppressed the effects of QDN in increasing ATP levels and decreasing ROS in lung cancer cells (Figure 6E,F). Mitochondrial ROS serve as a redox signalling molecule in apoptosis. We investigated the role of mitochondrial ROS in the anti‐cancer activity of QDN by utilizing the mitochondrial antioxidant MitoQ.^23^ MitoQ preconditioning partly attenuated the QDN‐induced ROS increase (Figure S3). These results provide additional evidence that QDN causes mitochondrial dysfunction and induces apoptosis via mitochondrial fragmentation.

### 3.6 QDN inhibited tumour growth in vivo

**FIGURE 7:**
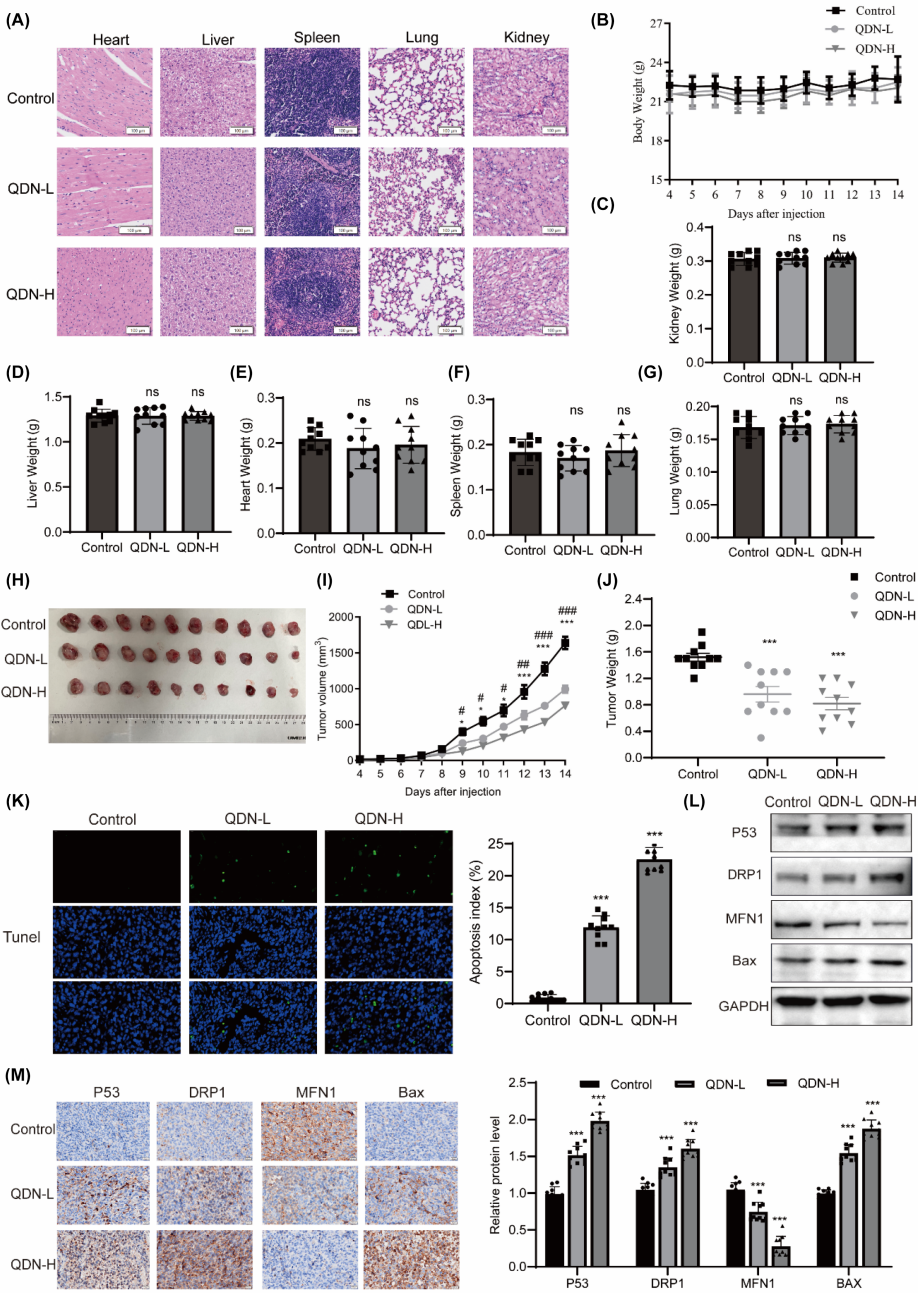
QDN inhibited tumour growth with acceptable safety in vivo. (A) HE‐stained pictures of the major organs: 100 μm. (B) Body weight and relative body weight changes. (C–G) Organ weights. (H) Gross images of tumours. (I) Tumour volumes. (J) Tumour weights. (K) TUNEL assay: 10 μm. (L) Protein expression of p53, DRP1, MFN1 and BAX in tumour. (M) Representative immunohistochemistry images of P53, DRP1, MFN1 and BAX: 20 μm. Data are mean ± SD of duplicate experiments (*n* = 10). Parametric variables were calculated using two‐way ANOVA. #*p* < 0.05, ##*p* < 0.01, ###*p* < 0.001, QDN‐L compared with control group, *p < 0.05, **p < 0.01, ***p < 0.001, ns, no significant difference, QDN‐H compared with control group. QDN‐H, high dose of QDN; QDN‐L, low dose of QDN.

Male C57 BL/6 mice were used to evaluate the safety after the oral administration of QDN. The mice were orally gavaged with QDN during the experiment, and the body weight, activity status and general appearance of each mouse were daily monitored during the experiment. The heart, liver, spleen, lung and kidney of the control animal and QDN‐treated mice were subjected to H&E staining to observe and compare the histological structures of organ sections under a microscope, and no significant histological changes were observed after the treatment (Figure 7A). There was no reduction in body weight after QDN treatments, respectively (Figure 7B). Furthermore, statistical analysis showed no significant difference in QDN‐treated mice based on a comparison with control mice (*p* > 0.05) concerning the major organ weights (Figure 7C–G). These findings preliminarily revealed the safety of QDN.

To probe the anti‐cancer effect of QDN in vivo, the LLC xenografts' subcutaneous xenograft tumour model was established. As shown in Figure 7H–J, the suppression of tumour growth by QDN was accompanied by a significant reduction in tumour volume and weight. TUNEL‐positive nuclei were also observed to be dose‐dependently increased (Figure 7K). In QDN‐treated LLC xenograft tumours, the protein levels of P53, DRP1 and Bax were increased, while the protein levels of MFN1 were decreased, as assessed by protein blotting (Figure 7L). Immunohistochemical analysis showed that QDN upregulated P53, DRP1 and Bax expression, and reduced MFN1 level (Figure 7M). We extracted mitochondria from the tumour and observed no significant change in the level of mitochondrial P53 (Figure S2G).

Consistent with in vitro experiments, these findings indicate that QDN inhibits NSCLC growth by inducing apoptosis and modulating mitochondrial fission.

## 4 DISCUSSION

Traditional Chinese medicine (TCM) has been widely practiced in China for thousands of years and has garnered increasing global attention.^24^, ^25^ The remarkable anti‐cancer properties of active ingredients derived from TCM, which exhibit minimal toxicity, offer a significant advantage. Our previous study has revealed that QDN, a TCM formula, exerts anti‐tumour effects by inducing apoptosis in lung cancer cells.^15^, ^17^, ^26^ In this study, our results suggest a close link between the inhibitory activity of QDN in lung cancer and energy metabolism as well as mitochondrial dynamics in lung cancer cells. QDN suppressed the proliferation, cloning, migration and metastasis of A549 and H460 cells in vitro and reduced tumour size in vivo with low side effects. Further analysis indicated that QDN induced anti‐tumour potency in promoting apoptosis and causing mitochondrial dysfunction via p53/DRP1‐mediated mitochondrial division.

Apoptosis, a programmed cell death, is considered one of the most effective strategies for cancer treatment.^27^, ^28^ Accumulating research has shown that balanced mitochondrial dynamics are strongly associated with intrinsic apoptotic pathways, enhancing mitochondrial division promotes apoptosis.^29^, ^30^ Extensive studies have illustrated that Chinese herbs and their active ingredients induced anti‐tumour effects by the mitochondrial apoptosis pathway.^31^ QDN induces NSCLC cell apoptosis by targeting mitochondrial fission through p53/DRP1 activation, together with impaired mitochondrial function characterized by decreased ATP production and ROS accumulation. Our finding indicates that QDN triggers mitochondrial dynamic imbalance to induce apoptosis, which might be a prospective strategy for lung cancer.

Mitochondrial dynamics is a crucial biodynamic process in cancer occurrence and progression.^20^ Mitochondria, a highly dynamic organelle, can continuously reshape their dynamic balance of form, function and number through division and fusion.^32^ Mitochondrial fragmentation from fission is tightly linked to apoptosis, and a balance shift in mitochondrial morphology to fission increases susceptibility to cell death.^33^ DRP1 dynamically regulates mitochondrial morphology, with its recruitment to the outer mitochondrial membrane being critical for mitochondrial fission.^34^ We observed that QDN enhanced mitochondrial fission, evidenced by increased mitochondrial fragments and enhanced levels of mitochondrial fission protein DRP1. Mdivi‐1, an inhibitor of mitochondrial division, effectively blocked QDN‐mediated mitochondrial division and apoptosis by treatment of NSCLC cells. In addition, tumour xenograft models indicated that QDN increased DRP1 expression and the pro‐apoptotic factor Bax in vivo. Thus, all the findings above supported that mitochondrial division enhancement may take a significant role in QDN‐induced apoptosis in NSCLC.

Structural and morphological changes in mitochondria carry important implications for mitochondrial function.^35^, ^36^ DRP1‐triggered mitochondrial fission causes a loss of MMP and damaged mitochondrial function, including a decrease in cellular ATP and ROS production.^37^, ^38^ DRP1 knockdown significantly inhibited mitochondrial fission, MMP loss, ATP depletion and apoptosis in breast cancer.^39^ Functional fusion of mitochondria acts as a countermeasure against metabolic damage maintains cellular integrity and prevents apoptosis. Mitochondrial fusion boosts ATP production, whereas reduced fusion leads to ROS accumulation and oxidative stress.^40^ Alterations in mitochondrial morphology have been identified as regulators of ROS production. The downregulation of AIM2 results in heightened expression of MFN1, thereby facilitating mitochondrial fusion and consequent diminishment of ROS levels.^41^ Moreover, elevated mitochondrial ROS can impact various redox signalling pathways. Further research is required to investigate whether redox signalling plays a role in QDN‐induced apoptosis. In the present study, QDN effects on mitochondrial morphology have been revealed. We further noted that QDN‐treated cells with significantly higher ROS levels and loss of mitochondrial membrane potential were accompanied by reduced ATP levels. Therefore, our data indicated that QDN‐mediated changes in mitochondrial morphology exerted a regulative effect on mitochondrial energy and function.

Previous studies have revealed that QDN exerts an anti‐tumour property by induction of apoptosis in several lung cancer cells, while the mechanism is not clear. We also found enhanced expression of p53 in QDN‐treated lung cancer cells, and further study is needed to explore whether p53 is involved in QDN‐induced apoptosis. The transcription factor p53 is believed to be involved in maintaining genomic integrity.^10^ The intrinsic mitochondrial apoptosis is induced by the epigenetic reactivation of p53, upregulating pro‐apoptotic and downregulating anti‐apoptotic genes.^42^ Pretreatment of NSCLC cells with the p53 inhibitor PFT‐α effectively blocked QDN‐mediated mitochondrial division and mitochondrial dysfunction. In addition, PFT‐α pretreatment prevented QDN‐induced apoptosis. With a lentiviral vector system, we found that p53 overexpression not only dramatically increased QDN‐induced apoptosis but also enhanced mitochondrial division and mitochondrial dysfunction. Our study established the link between QDN treatment of p53‐mediated apoptosis and mitochondrial morphology and function. QDN may facilitate mitochondrial division and mitochondrial dysfunction via tumour suppressor p53 to trigger NSCLC apoptosis.

In conclusion, QDN effectively induces apoptosis in lung cancer cells and is closely related to mitochondrial dynamics. QDN affects the mitochondrial structure, function and metabolism through a p53/DRP1‐mediated increase in mitochondrial fission, which ultimately induces lung cancer cell apoptosis. Therefore, the present work offered a new perspective on NSCLC treatment.

## AUTHOR CONTRIBUTIONS

**Rongzhen Ding:** Supervision (equal); validation (lead); writing – original draft (lead). **Yichao Wang:** Data curation (equal); writing – original draft (equal). **Ling Xu:** Funding acquisition (equal); resources (equal); validation (equal). **Shuliu Sang:** Resources (equal); software (equal). **Guanjin Wu:** Investigation (equal); visualization (equal). **Wenxiao Yang:** Methodology (equal); supervision (equal). **Yilu Zhang:** Data curation (equal); investigation (equal). **Chengyan Wang:** Software (equal); visualization (equal). **Ao Qi:** Formal analysis (equal); writing – original draft (equal). **Haiping Xie:** Validation (equal); writing – review and editing (equal). **Yi Liu:** Data curation (equal); formal analysis (equal). **Aiguo Dai:** Supervision (equal); writing – review and editing (equal). **Lijing Jiao:** Project administration (equal); software (equal); supervision (equal); writing – review and editing (equal).

## CONFLICT OF INTEREST STATEMENT

The authors declare no competing interest.

## Supporting information


Data S1.

## Data Availability

The data supporting the findings of this study are available within the article.

## References

[1] Siegel RL , Miller KD , Fuchs HE , Jemal A . Cancer statistics, 2022. CA Cancer J Clin. 2022;72:7‐33. 35020204 10.3322/caac.21708

[2] D'Arcy MS . Cell death: a review of the major forms of apoptosis, necrosis and autophagy. Cell Biol Int. 2019;43:582‐592. 30958602 10.1002/cbin.11137

[3] Filograna R , Mennuni M , Alsina D , Larsson N‐G . Mitochondrial DNA copy number in human disease: the more the better? FEBS Lett. 2021;595:976‐1002. 33314045 10.1002/1873-3468.14021 PMC8247411

[4] Wei W , Chinnery PF . Cracking the enigma of mitochondrial‐DNA variants and cancer. Nat Metab. 2020;2:221‐222. 32694774 10.1038/s42255-020-0180-2

[5] Adebayo M , Singh S , Singh AP , Dasgupta S . Mitochondrial fusion and fission: the fine‐tune balance for cellular homeostasis. FASEB J. 2021;35:e21620. 34048084 10.1096/fj.202100067R PMC8415099

[6] Dasgupta A , Chen KH , Lima PDA , et al. PINK1‐induced phosphorylation of mitofusin 2 at serine 442 causes its proteasomal degradation and promotes cell proliferation in lung cancer and pulmonary arterial hypertension. FASEB J. 2021;35:e21771. 34275172 10.1096/fj.202100361R PMC8294132

[7] Jackson M , Serada N , Sheehan M , et al. Mitochondrial genome and functional defects in osteosarcoma are associated with their aggressive phenotype. PLoS One. 2018;13:e0209489. 30576337 10.1371/journal.pone.0209489 PMC6303035

[8] Chen J , Zhang L , Yu X , et al. Clinical application of plasma mitochondrial DNA content in patients with lung cancer. Oncol Lett. 2018;16:7074‐7081. 30546441 10.3892/ol.2018.9515 PMC6256833

[9] Ko YS , Kang H , Bae JA , et al. New strategy for suppressing the growth of lung cancer cells harboring mutations in the ATP‐binding region of EGFR by targeting the molecular motor MYO1D. Clin Transl Med. 2021;11:e515. 34459138 10.1002/ctm2.515 PMC8343539

[10] Marei HE , Althani A , Afifi N , et al. p53 signaling in cancer progression and therapy. Cancer Cell Int. 2021;21:703. 34952583 10.1186/s12935-021-02396-8 PMC8709944

[11] Hafner A , Bulyk ML , Jambhekar A , Lahav G . The multiple mechanisms that regulate p53 activity and cell fate. Nat Rev Mol Cell Biol. 2019;20:199‐210. 30824861 10.1038/s41580-019-0110-x

[12] Warren CFA , Wong‐Brown MW , Bowden NA . BCL‐2 family isoforms in apoptosis and cancer. Cell Death Dis. 2019;10:177. 30792387 10.1038/s41419-019-1407-6 PMC6384907

[13] Aziz YMA , Lotfy G , Said MM , et al. Design, synthesis, chemical and biochemical insights into novel hybrid Spirooxindole‐based p53‐MDM2 inhibitors with potential Bcl2 signaling attenuation. Front Chem. 2021;9:735236. 34970530 10.3389/fchem.2021.735236 PMC8713455

[14] Yao H , Mi S , Gong W , et al. Anti‐apoptosis proteins Mcl‐1 and Bcl‐xL have different p53‐binding profiles. Biochemistry. 2013;52:6324‐6334. 23977882 10.1021/bi400690m

[15] Zheng T , Que Z , Jiao L , et al. Herbal formula YYJD inhibits tumor growth by inducing cell cycle arrest and senescence in lung cancer. Sci Rep. 2017;7:4984. 28694520 10.1038/s41598-017-05146-x PMC5504005

[16] Teng JF , Mei QB , Zhou XG , et al. Polyphyllin VI induces Caspase‐1‐mediated Pyroptosis via the induction of ROS/NF‐κB/NLRP3/GSDMD signal Axis in non‐small cell lung cancer. Cancers (Basel). 2020;12:12. 10.3390/cancers12010193 PMC7017302 31941010

[17] Yang W , Kang Y , Zhao Q , et al. Herbal formula Yangyinjiedu induces lung cancer cell apoptosis via activation of early growth response 1. J Cell Mol Med. 2019;23:6193‐6202. 31237749 10.1111/jcmm.14501 PMC6714142

[18] Valente AJ , Maddalena LA , Robb EL , Moradi F , Stuart JA . A simple ImageJ macro tool for analyzing mitochondrial network morphology in mammalian cell culture. Acta Histochem. 2017;119:315‐326. 28314612 10.1016/j.acthis.2017.03.001

[19] Nayak A , Warrier NM , Kumar P . Cancer stem cells and the tumor microenvironment: targeting the critical crosstalk through Nanocarrier systems. Stem Cell Rev Rep. 2022;18:2209‐2233. 35876959 10.1007/s12015-022-10426-9 PMC9489588

[20] Kumar S , Ashraf R , Aparna CK . Mitochondrial dynamics regulators: implications for therapeutic intervention in cancer. Cell Biol Toxicol. 2022;38:377‐406. 34661828 10.1007/s10565-021-09662-5

[21] Praharaj PP , Patro BS , Bhutia SK . Dysregulation of mitophagy and mitochondrial homeostasis in cancer stem cells: novel mechanism for anti‐cancer stem cell‐targeted cancer therapy. Br J Pharmacol. 2022;179:5015‐5035. 33527371 10.1111/bph.15401

[22] Zhang J , Qiao W , Luo Y . Mitochondrial quality control proteases and their modulation for cancer therapy. Med Res Rev. 2022;43:399‐436. 36208112 10.1002/med.21929

[23] Wang B , Wang Y , Zhang J , et al. ROS‐induced lipid peroxidation modulates cell death outcome: mechanisms behind apoptosis, autophagy, and ferroptosis. Arch Toxicol. 2023;97:1439‐1451. 37127681 10.1007/s00204-023-03476-6

[24] Tang Q , Wang X , Zhou Q , et al. Fuzheng Kang‐Ai inhibits NSCLC cell proliferation via regulating hsa_circ_0048091/hsa‐miR‐378g/ARRDC3 pathway. Phytomedicine. 2023;114:154819. 37062135 10.1016/j.phymed.2023.154819

[25] Song M , Qian C , Zhang T , et al. Salvia mitiorrhiza Bunge aqueous extract attenuates infiltration of tumor‐associated macrophages and potentiates anti‐PD‐L1 immunotherapy in colorectal cancer through modulating Cox2/PGE2 cascade. J Ethnopharmacol. 2023;316:116735. 37286115 10.1016/j.jep.2023.116735

[26] Lu J , Chen J , Xu N , et al. Activation of AIFM2 enhances apoptosis of human lung cancer cells undergoing toxicological stress. Toxicol Lett. 2016;258:227‐236. 27392435 10.1016/j.toxlet.2016.07.002

[27] Singh P , Lim B . Targeting apoptosis in cancer. Curr Oncol Rep. 2022;24:273‐284. 35113355 10.1007/s11912-022-01199-y

[28] Westaby D , Jimenez‐Vacas JM , Padilha A , et al. Targeting the intrinsic apoptosis pathway: a window of opportunity for prostate cancer. Cancers (Basel). 2021;14:14. 35008216 10.3390/cancers14010051 PMC8750516

[29] Li K , van Delft MF , Dewson G . Too much death can kill you: inhibiting intrinsic apoptosis to treat disease. EMBO J. 2021;40:e107341. 34037273 10.15252/embj.2020107341 PMC8280825

[30] Jenner A , Peña‐Blanco A , Salvador‐Gallego R , et al. DRP1 interacts directly with BAX to induce its activation and apoptosis. EMBO J. 2022;41:e108587. 35023587 10.15252/embj.2021108587 PMC9016351

[31] Xia D , Li W , Tang C , Jiang J . Astragaloside IV, as a potential anticancer agent. Front Pharmacol. 2023;14:1065505. 36874003 10.3389/fphar.2023.1065505 PMC9981805

[32] Tilokani L , Nagashima S , Paupe V , Prudent J . Mitochondrial dynamics: overview of molecular mechanisms. Essays Biochem. 2018;62:341‐360. 30030364 10.1042/EBC20170104 PMC6056715

[33] Kim D , Sankaramoorthy A , Roy S . Downregulation of Drp1 and Fis1 inhibits mitochondrial fission and prevents high glucose‐induced apoptosis in retinal endothelial cells. Cells. 2020;9:1662. 32664237 10.3390/cells9071662 PMC7407825

[34] Rahmani S , Roohbakhsh A , Karimi G . Inhibition of Drp1‐dependent mitochondrial fission by natural compounds as a therapeutic strategy for organ injuries. Pharmacol Res. 2023;188:106672. 36690165 10.1016/j.phrs.2023.106672

[35] Yapa NMB , Lisnyak V , Reljic B , Ryan MT . Mitochondrial dynamics in health and disease. FEBS Lett. 2021;595:1184‐1204. 33742459 10.1002/1873-3468.14077

[36] Wang M , Wei R , Li G , et al. SUMOylation of SYNJ2BP‐COX16 promotes breast cancer progression through DRP1‐mediated mitochondrial fission. Cancer Lett. 2022;547:215871. 35998797 10.1016/j.canlet.2022.215871

[37] Deng X , Liu J , Liu L , Sun X , Huang J , Dong J . Drp1‐mediated mitochondrial fission contributes to baicalein‐induced apoptosis and autophagy in lung cancer via activation of AMPK signaling pathway. Int J Biol Sci. 2020;16:1403‐1416. 32210728 10.7150/ijbs.41768 PMC7085231

[38] Fan K , Ding X , Zang Z , et al. Drp1‐mediated mitochondrial metabolic dysfunction inhibits the tumor growth of pituitary adenomas. Oxid Med Cell Longev. 2022;2022:5652586. 35368865 10.1155/2022/5652586 PMC8967574

[39] Tang Q , Liu W , Zhang Q , et al. Dynamin‐related protein 1‐mediated mitochondrial fission contributes to IR‐783‐induced apoptosis in human breast cancer cells. J Cell Mol Med. 2018;22:4474‐4485. 29993201 10.1111/jcmm.13749 PMC6111821

[40] Noh S , Phorl S , Naskar R , et al. p32/C1QBP regulates OMA1‐dependent proteolytic processing of OPA1 to maintain mitochondrial connectivity related to mitochondrial dysfunction and apoptosis. Sci Rep. 2020;10:10618. 32606429 10.1038/s41598-020-67457-w PMC7327069

[41] Qi M , Dai D , Liu J , et al. AIM2 promotes the development of non‐small cell lung cancer by modulating mitochondrial dynamics. Oncogene. 2020;39:2707‐2723. 32005973 10.1038/s41388-020-1176-9

[42] Sanaei M , Kavoosi F . Effect of Valproic acid on the class I histone deacetylase 1, 2 and 3, tumor suppressor genes p21WAF1/CIP1 and p53, and intrinsic mitochondrial apoptotic pathway, pro‐ (Bax, Bak, and Bim) and anti‐ (Bcl‐2, Bcl‐xL, and Mcl‐1) apoptotic genes expression, cell viability, and apoptosis induction in hepatocellular carcinoma HepG2 cell line. Asian Pac J Cancer Prev. 2021;22:89‐95. 10.31557/APJCP.2021.22.S1.89 33576217

