# Supplementary material for: QiDongNing induces lung cancer cell apoptosis via triggering P53/DRP1‐mediated mitochondrial fission
Source: J Cell Mol Med. 2024 Apr 29;28(9):e18353. doi: 10.1111/jcmm.18353 (PMC11057058; doi:10.1111/jcmm.18353)

**Supplement table 1**

Active ingredients and activity prediction of QDN

| Number | tR(min) | MFG Formula | Theoretical value | Measured value | Error  (ppm) | Positiv ion mode | Negative ion mode | Compound Label | Sourcce |
| --- | --- | --- | --- | --- | --- | --- | --- | --- | --- |
| 1 | 0.94 | C_6_H_14_N_4_O_2_ | 175.1190 | 175.1189 | -0.40 | [M+H]+ | [M-H]- | L(+)-Arginine | Astragalus, Paris polyphylla |
| 2 | 1.00 | C_6_H_13_N_3_O_3_ | 176.1030 | 176.1027 | -1.30 | [M+H]+ |  | Citrulline | Astragalus, Radix Ophiopogonis |
| 3 | 1.04 | C_5_H_11_NO_2_ | 118.0863 | 118.0860 | -2.10 | [M+H]+ |  | Betaine | Paris polyphylla |
| 4 | 1.08 | C_5_H_9_NO_2_ | 116.0710 | 116.0702 | -3.20 | [M+H]+ |  | Proline | Astragalus, Radix Ophiopogonis |
| 5 | 1.19 | C_6_H_6_O_3_ | 127.0390 | 127.0385 | -3.80 | [M+H]+ |  | 5-Hydroxymethylfurfural | Glossy Privet Fruit |
| 6 | 1.33 | C_6_H_8_O_7_ | 191.0200 | 191.0203 | 3.00 |  | [M-H]- | Citric acid | Fiveleaf Gynostemma. |
| 7 | 1.40 | C_5_H_4_N_4_O | 137.0460 | 137.0454 | -2.50 | [M+H]+ |  | 6-Hydroxypurine | Astragalus |
| 8 | 1.73 | C_9_H_13_N_3_O_5_ | 244.0930 | 244.0928 | 0.20 | [M+H]+ |  | Cytidine | Astragalus |
| 9 | 1.75 | C_5_H_5_N_5_ | 136.0620 | 136.0615 | -2.20 | [M+H]+ |  | Adenine | Astragalus |
| 10 | 2.27 | C_5_H_7_N_O3_ | 128.0350 | 128.0353 | 0.10 |  | [M-H]- | pyroglutamte | Astragalus, Radix Ophiopogonis |
| 11 | 2.82 | C_24_H_42_O_21_ | 665.2150 | 665.2146 | 0.00 |  | [M-H]- | Stachyose | Radix Ophiopogonis |
| 12 | 2.91 | C_6_H_13_NO_2_ | 130.0870 | 130.0872 | -1.00 |  | [M-H]- | Isoleucine | Astragalus, Paris polyphylla, Radix Ophiopogonis |
| 13 | 3.02 | C_12_H_22_O_11_ | 341.1090 | 341.1087 | -0.60 |  | [M-H]- | Sucrose | Glossy Privet Fruit |
| 14 | 3.06 | C_9_H_11_NO_3_ | 180.0670 | 180.0668 | 0.90 |  | [M-H]- | Tyrosine | Radix Ophiopogonis, Astragalus, Radix Ophiopogonis |
| 15 | 3.34 | C_9_H_12_N_2_O_6_ | 243.0620 | 243.0624 | 0.80 |  | [M-H]- | Uridine | Astragalus |
| 16 | 3.49 | C_10_H_13_N_5_O_4_ | 266.0890 | 266.0897 | 0.90 |  | [M-H]- | Adenosine | Paris polyphylla |
| 17 | 3.84 | C_7_H_6_O_4_ | 153.0190 | 153.0195 | 0.80 |  | [M-H]- | Patulin | Astragalus |
| 18 | 4.84 | C_5_H_8_O_4_ | 131.0350 | 131.0350 | -0.10 |  | [M-H]- | Ethylmalonic acid | Paris polyphylla |
| 19 | 5.02 | C_10_H_12_N_4_O_5_ | 267.0730 | 267.0736 | 0.60 |  | [M-H]- | Inosine | Paris polyphylla |
| 20 | 5.44 | C_5_H_8_O_4_ | 131.0350 | 131.0351 | 0.60 |  | [M-H]- | Glutaric acid | Paris polyphylla |
| 21 | 5.73 | C_10_H_13_N_5_O_5_ | 282.0840 | 282.0845 | 0.30 |  | [M-H]- | guanosine | Paris polyphylla |
| 22 | 6.33 | C_9_H_17_NO_5_ | 218.1030 | 218.1039 | 2.40 |  | [M-H]- | Pantothenic acid | Paris polyphylla |
| 23 | 7.07 | C_14_H_20_O_7_ | 345.1190 | 345.1194 | 0.90 |  | [M+COOH]- | Salidroside | Glossy Privet Fruit |
| 24 | 7.77 | C_16_H_18_O_9_ | 355.1020 | 355.1030 | 1.80 | [M+H]+ | [M-H]- | Chlorogenic acid | Astragalus, Fiveleaf Gynostemma. |
| 25 | 8.10 | C_10_H_12_O_2_ | 165.0910 | 165.0918 | 4.60 | [M+H]+ |  | Eugenol | Radix Ophiopogonis |
| 26 | 8.16 | C_10_H_16_O | 153.1270 | 153.1274 | -0.20 | [M+H]+ |  | (E,E)- 2,4-decadienal | Astragalus |
| 27 | 8.19 | C_9_H_6_O_4_ | 179.0340 | 179.0339 | 0.30 | [M+H]+ | [M-H]- | Daphnetin | Radix Ophiopogonis |
| 28 | 8.25 | C_8_H_8_O_4_ | 169.0500 | 169.0497 | 0.90 | [M+H]+ | [M-H]- | Vanillic acid | Radix Ophiopogonis |
| 29 | 8.32 | C_15_H_10_O_5_ | 271.0600 | 271.0601 | 0.10 | [M+H]+ |  | Genistein | Astragalus |
| 30 | 8.36 | C_35_H_46_O_20_ | 804.2920 | 804.2919 | -0.20 | [M+NH4]+ | [M-H]- | Echinacoside | Glossy Privet Fruit |
| 31 | 8.47 | C_9_H_11_NO_2_ | 166.0860 | 166.0860 | -1.80 | [M+H]+ |  | Phenylalanine | Astragalus, Paris polyphylla |
| 32 | 8.70 | C_7_H_12_O_4_ | 159.0660 | 159.0664 | 0.80 |  | [M-H]- | Pimelic acid | Radix Ophiopogonis |
| 33 | 8.87 | C_17_H_24_O_11_ | 403.1250 | 403.1242 | -0.90 |  | [M-H]- | Deacetyl asperulosidic acid methyl ester | Glossy Privet Fruit |
| 34 | 9.18 | C_28_H_34_O_15_ | 609.1820 | 609.2034 |  |  | [M-H]- | Hesperidin | Astragalus, Radix Ophiopogonis |
| 35 | 9.28 | C_9_H_8_O_3_ | 163.0400 | 163.0399 | -0.90 |  | [M-H]- | 3-Coumaric acid | Astragalus |
| 36 | 9.43 | C_27_H_44_O_7_ | 481.3160 | 481.3164 | 0.90 | [M+H]+ | [M-H]- | β-Ecdysone | Paris polyphylla |
| 37 | 9.46 | C_31_H_42_O_18_ | 701.2300 | 701.2302 | 0.50 |  | [M-H]- | Neonuezhenide | Glossy Privet Fruit |
| 38 | 9.53 | C_31_H_42_O_17_ | 685.2350 | 685.2345 | -0.60 |  | [M-H]- | Specnuezhenide | Glossy Privet Fruit |
| 39 | 9.67 | C_10_H_10_O_4_ | 195.0650 | 195.0651 | -0.40 | [M+H]+ |  | Ferulic Acid | Radix Ophiopogonis |
| 40 | 9.68 | C_21_H_20_O_10_ | 433.1130 | 433.1126 | -0.80 | [M+H]+ |  | Apigenin-7-glucoside | Glossy Privet Fruit, Fiveleaf Gynostemma. |
| 41 | 9.75 | C_8_H_8_O | 121.0650 | 121.0648 | 0.10 | [M+H]+ |  | Phenylacetaldehyde | Radix Ophiopogonis |
| 42 | 9.81 | C_9_H_8_O | 133.0650 | 133.0644 | -2.70 | [M+H]+ |  | Cinnamaldehyde | Radix Ophiopogonis |
| 43 | 9.89 | C_31_H_42_O_17_ | 731.2400 | 731.2399 | -0.70 | [M+COOH]- |  | Specnuezhenide | Glossy Privet Fruit |
| 44 | 9.93 | C_21_H_20_O_11_ | 449.1080 | 449.1082 | -0.10 | [M+H]+ | [M-H]- | Quercitrin | Glossy Privet Fruit |
| 45 | 10.09 | C_21_H_20_O_10_ | 433.1130 | 433.1129 | -0.10 | [M+H]+ |  | Naringenin-7-glucoside | Fiveleaf Gynostemma. |
| 46 | 10.11 | C_21_H_20_O_12_ | 465.1030 | 465.1027 | -0.10 | [M+H]+ |  | Hyperin | Astragalus, Fiveleaf Gynostemma. |
| 47 | 10.12 | C_22_H_24_O_10_ | 449.1440 | 449.1444 | 0.30 | [M+H]+ |  | Licoagroside D | Astragalus |
| 48 | 10.13 | C_21_H_18_O_11_ | 447.0920 | 447.0926 | 0.90 | [M+H]+ |  | Apigenin 7-O-beta-D-glucuronide | Astragalus, Fiveleaf Gynostemma., Radix Ophiopogonis |
| 49 | 10.34 | C_9_H_16_O_4_ | 187.0980 | 187.0977 | 0.60 |  | [M-H]- | Azelaic acid | Radix Ophiopogonis |
| 50 | 10.43 | C_15_H_10_O_5_ | 269.0460 | 269.0455 | -0.20 |  | [M-H]- | Apigenin | Astragalus, Fiveleaf Gynostemma., Radix Ophiopogonis |
| 51 | 10.48 | C_22_H_22_O_10_ | 447.1290 | 447.1287 | 0.20 | [M+H]+ |  | Diosmetin-7-O-D-glucopyranoside | Astragalus, Radix Ophiopogonis |
| 52 | 10.51 | C_16_H_12_O_6_ | 301.0710 | 301.0708 | 0.40 | [M+H]+ |  | rhamnocitrin | Astragalus |
| 53 | 10.56 | C_15_H_10_O_6_ | 287.0550 | 287.0550 | -0.10 | [M+H]+ |  | Kaempferol | Glossy Privet Fruit, Astragalus, Paris polyphylla, Fiveleaf Gynostemma. |
| 54 | 10.57 | C_28_H_32_O_16_ | 625.1760 | 625.1769 | 1.00 | [M+H]+ | [M-H]- | Isorhamnetin-3-O-neohespeidoside | Paris polyphylla |
| 55 | 10.61 | C_31_H_42_O_17_ | 685.2350 | 685.2347 | -0.40 |  | [M-H]- | Nuezhenide | Glossy Privet Fruit |
| 56 | 10.68 | C_7_H_6_O_3_ | 137.0240 | 137.0245 | 0.60 |  |  | salicylic acid | Radix Ophiopogonis |
| 57 | 10.70 | C_54_H_92_O_23_ | 1153.6010 | 1153.6008 |  |  | [M+COOH]- | Ginsenoside Rb1 | Fiveleaf Gynostemma. |
| 58 | 10.74 | C_22_H_22_O_9_ | 431.1340 | 431.1341 | 0.90 | [M+H]+ | [M-H]- | Ononin | Astragalus |
| 59 | 11.26 | C_23_H_28_O_10_ | 465.1760 | 465.1759 | 0.90 | [M+H]+ | [M-H]- | Isomucronulatol-7-O-glucoside | Astragalus |
| 60 | 11.28 | C_22_H_22_O_11_ | 463.1230 | 285.0761 | 0.30 | [M+H]+ |  | Pratensein-7-O-glucoside | Astragalus |
| 61 | 11.59 | C_16_H_12_O_5_ | 285.0760 | 447.1284 | 1.40 | [M+H]+ | [M-H]- | Calycosin | Astragalus |
| 62 | 11.73 | C_22_H_22_O_10_ | 447.1290 | 623.1977 | -0.30 | [M+H]+ |  | calycosin-7-o-glucoside | Astragalus |
| 63 | 11.77 | C_29_H_34_O_15_ | 623.1970 | 149.0598 | 1.00 | [M+H]+ | [M-H]- | pectolinarin | Fiveleaf Gynostemma. |
| 64 | 11.91 | C_9_H_8_O_2_ | 149.0600 | 829.4597 | 0.40 | [M+H]+ |  | cinnamic acid | Radix Ophiopogonis |
| 65 | 12.08 | C_41_H_68_O_14_ | 829.4590 | 845.4900 | 0.80 |  | [M+COOH]- | Astragaloside Ⅳ | Astragalus |
| 66 | 12.23 | C_42_H_72_O_14_ | 845.4900 | 215.1291 | -0.50 |  | [M+COOH]- | gynoside XXX | Fiveleaf Gynostemma. |
| 67 | 12.26 | C_11_H_20_O_4_ | 215.1290 | 287.0550 | 0.80 |  | [M-H]- | Undecanedioic acid | Radix Ophiopogonis |
| 68 | 12.50 | C_15_H_10_O_6_ | 287.0550 | 317.0658 | 0.60 | [M+H]+ | [M-H]- | Luteolin | Glossy Privet Fruit |
| 69 | 12.67 | C_16_H_12_O_7_ | 317.0660 | 991.5474 | 0.50 | [M+H]+ | [M-H]- | Isorhamnetin | Astragalus, Fiveleaf Gynostemma. |
| 70 | 12.88 | C_48_H_82_O_18_ | 991.5480 | 269.0812 | -0.90 |  | [M+COOH]- | Ginsenoside Rd | Fiveleaf Gynostemma. |
| 71 | 13.26 | C_16_H_12_O_4_ | 269.0810 | 269.0812 | 1.20 | [M+H]+ | [M-H]- | Formononetin | Astragalus |
| 72 | 13.46 | C_17_H_16_O_5_ | 301.1070 | 283.0237 | -0.20 | [M+H]+ |  | 3-Hydroxy-9,10-Dimethoxypterocarpan | Astragalus |
| 73 | 13.70 | C_15_H_8_O_6_ | 283.0250 | 243.1605 | -4.00 |  | [M-H]- | Rhein | Radix Ophiopogonis |
| 74 | 13.80 | C_13_H_24_O_4_ | 243.1600 | 871.4698 | 1.30 |  | [M-H]- | 1,11-Undecanedicarboxylic acid | Radix Ophiopogonis |
| 75 | 13.81 | C_43_H_70_O_15_ | 871.4700 | 871.4698 | 0.20 |  | [M+COOH]- | Astragaloside Ⅱ | Astragalus |
| 76 | 13.89 | C_45_H_72_O_16_ | 913.4800 | 871.4699 | -2.30 |  | [M+COOH]- | Dioscin | Radix Ophiopogonis |
| 77 | 14.00 | C_44_H_70_O_17_ | 871.4690 | 845.4914 | 0.70 | [M+H]+ | [M-H]- | Liriope muscari baily saponins C | Radix Ophiopogonis |
| 78 | 14.17 | C_42_H_72_O_14_ | 845.4900 | 756.4541 | 1.20 |  | [M+COOH]- | Ginsenoside Rf | Fiveleaf Gynostemma. |
| 79 | 14.29 | C_39_H_62_O_13_ | 756.4530 | 301.0707 |  | [M+NH4]+ |  | Polyphyllin VI | Paris polyphylla |
| 80 | 14.37 | C_16_H_12_O_6_ | 301.0710 | 455.3521 | 0.30 | [M+H]+ | [M-H]- | kaempferide | Glossy Privet Fruit |
| 81 | 14.53 | C_30_H_46_O_3_ | 455.3520 | 329.0669 | 0.30 | [M+H]+ |  | Betulonicacid | Glossy Privet Fruit |
| 82 | 14.59 | C_17_H_14_O_7_ | 329.0670 | 329.0669 | 0.80 |  | [M-H]- | Ombuin | Fiveleaf Gynostemma. |
| 83 | 14.85 | C_45_H_72_O_16_ | 913.4800 | 913.4806 | 0.30 |  | [M+COOH]- | Astragaloside I | Astragalus |
| 84 | 14.93 | C_30_H_50_O_2_ | 443.3880 | 443.3884 |  | [M+H]+ |  | Betulin | Glossy Privet Fruit |
| 85 | 14.96 | C_42_H_72_O_13_ | 829.4950 | 829.4956 | 0.10 |  | [M+COOH]- | Ginsenoside Rg2 | Fiveleaf Gynostemma. |
| 86 | 15.13 | C_16_H_30_O_4_ | 285.2070 | 285.2071 | -0.10 |  | [M-H]- | Hexadecanedioic acid | Radix Ophiopogonis |
| 87 | 15.42 | C_27_H_42_O_4_ | 431.3160 | 431.3157 | 0.20 | [M+H]+ |  | Ruscogenin | Radix Ophiopogonis |
| 88 | 15.62 | C_36_H_62_O_9_ | 683.4380 | 683.4374 | -0.20 |  | [M+COOH]- | Ginsenoside Rh1 | Fiveleaf Gynostemma. |
| 89 | 15.66 | C_45_H_72_O_17_ | 929.4750 | 929.4759 | 0.80 | [M+H]+ | [M-H]- | Gracillin | Paris polyphylla |
| 90 | 15.76 | C_44_H_70_O_16_ | 853.4590 | 853.4599 | 1.00 |  | [M-H]- | Polyphyllin I | Paris polyphylla |
| 91 | 15.92 | C_18_H_32_O_2_ | 281.2480 | 281.2477 | 0.80 | [M+H]+ |  | linoleic acid | Radix Ophiopogonis |
| 92 | 16.04 | C_48_H_76_O_19_ | 955.4910 | 955.4911 | 0.30 |  | [M-H]- | Ginsenoside-Ro | Fiveleaf Gynostemma. |
| 93 | 16.25 | C_39_H_62_O_12_ | 767.4220 | 767.4225 | 0.20 | [M+H]+ | [M-H]- | Liriopesides B | Radix Ophiopogonis |
| 94 | 16.26 | C_16_H_30_O_2_ | 255.2320 | 255.2321 | 0.80 | [M+H]+ |  | palmitoleate | Astragalus, Radix Ophiopogonis |
| 95 | 16.58 | C_36_H_62_O_8_ | 667.4430 | 667.4413 | -2.00 |  | [M+COOH]- | (S)-Ginsenoside Rh2 | Fiveleaf Gynostemma. |
| 96 | 16.72 | C_27_H_42_O_3_ | 415.3210 | 415.3212 | 1.20 | [M+H]+ |  | Diosgenin | Radix Ophiopogonis |
| 97 | 17.07 | C_30_H_48_O_4_ | 471.3480 | 471.3483 | -1.00 |  | [M-H]- | 2α-Hydroxyursolic acid | Glossy Privet Fruit |
| 98 | 19.60 | C_27_H_30_O_16_ | 609.1460 | 609.1465 | 0.60 |  | [M-H]- | Rutin | Glossy Privet Fruit, Astragalus, Fiveleaf Gynostemma. |

**Supplement table 2**

| Cell lines | QDN |
| --- | --- |
| A549 | 469.5 |
| H460 | 651.3 |
| LLC | 551.5 |

The IC_50_ of QDN in lung cancer cells (μg/ml)

**Supplement figure 1**

#### Total ion chromatogram for QDN after UPLC-Q-TOF-MS/MS separation

**a** Total composition chromatogram (TCC) in positive ionization mode. **b** TCC in negative ionization mode.

**
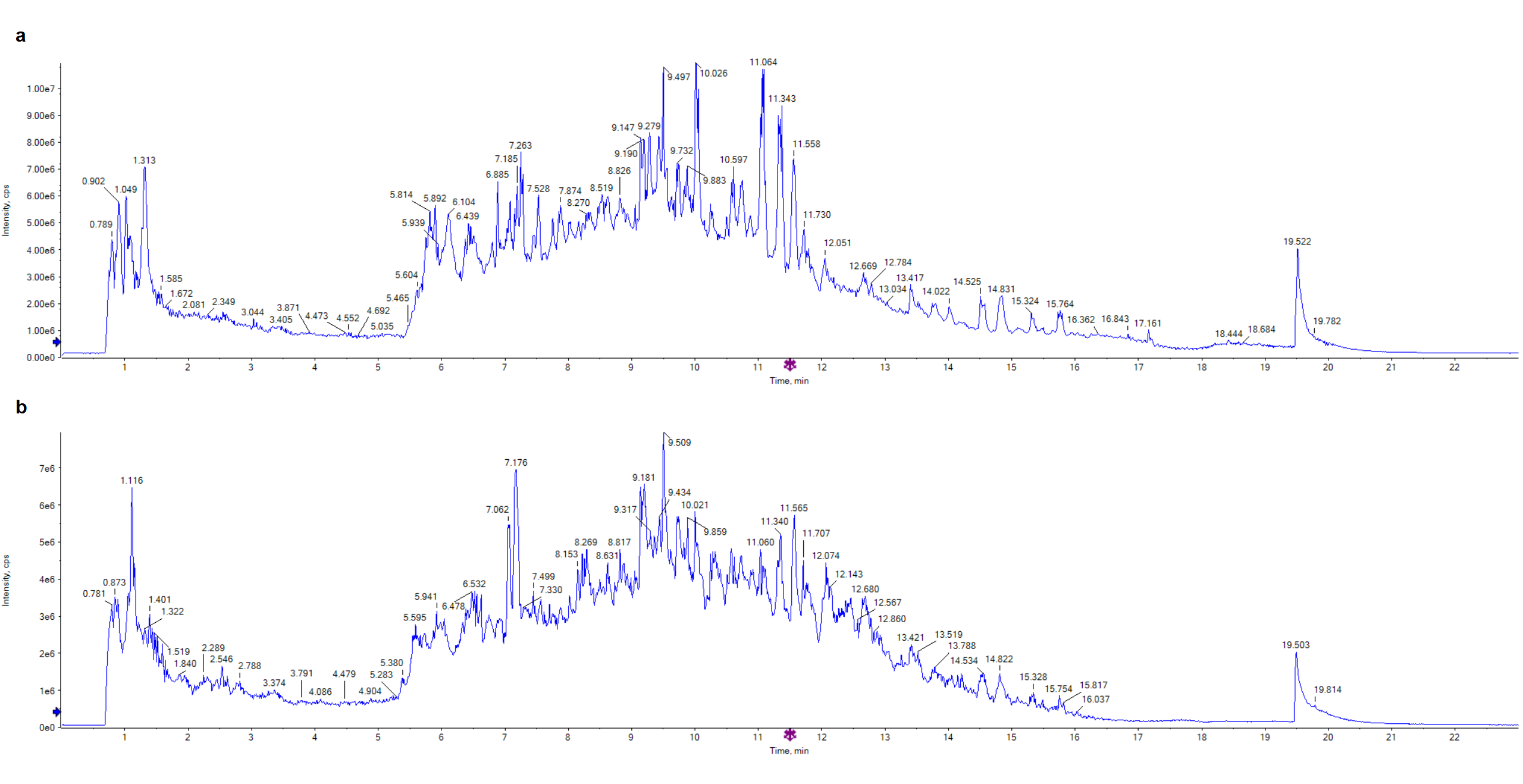
**

**Supplement figure 2**

#### The effect of QDN on necroptosis and mitochondrial p53

**a** Cell inhibition ratio in A549 cells after QDN administration for 48 h with or without Necrostatin-1 pretreatment. **b** Protein expression of RIPK1 in A549 cells. **c** Cell inhibition ratio in NCI-H460 cells after QDN administration for 48 h with or without Necrostatin-1 pretreatment. **d** Protein expression of RIPK1 in NCI-H460 cells. **e** mitochondrial p53 in A549 cells. **f** mitochondrial p53 in NCI-H460 cells. **g** mitochondrial p53 in tumor. Data are mean ± SD of duplicate experiments (n=3). Parametric variables were calculated using One-way ANOVA. ns, no significant difference. QDN-L: low dose of QDN, QDN-H: high dose of QDN.


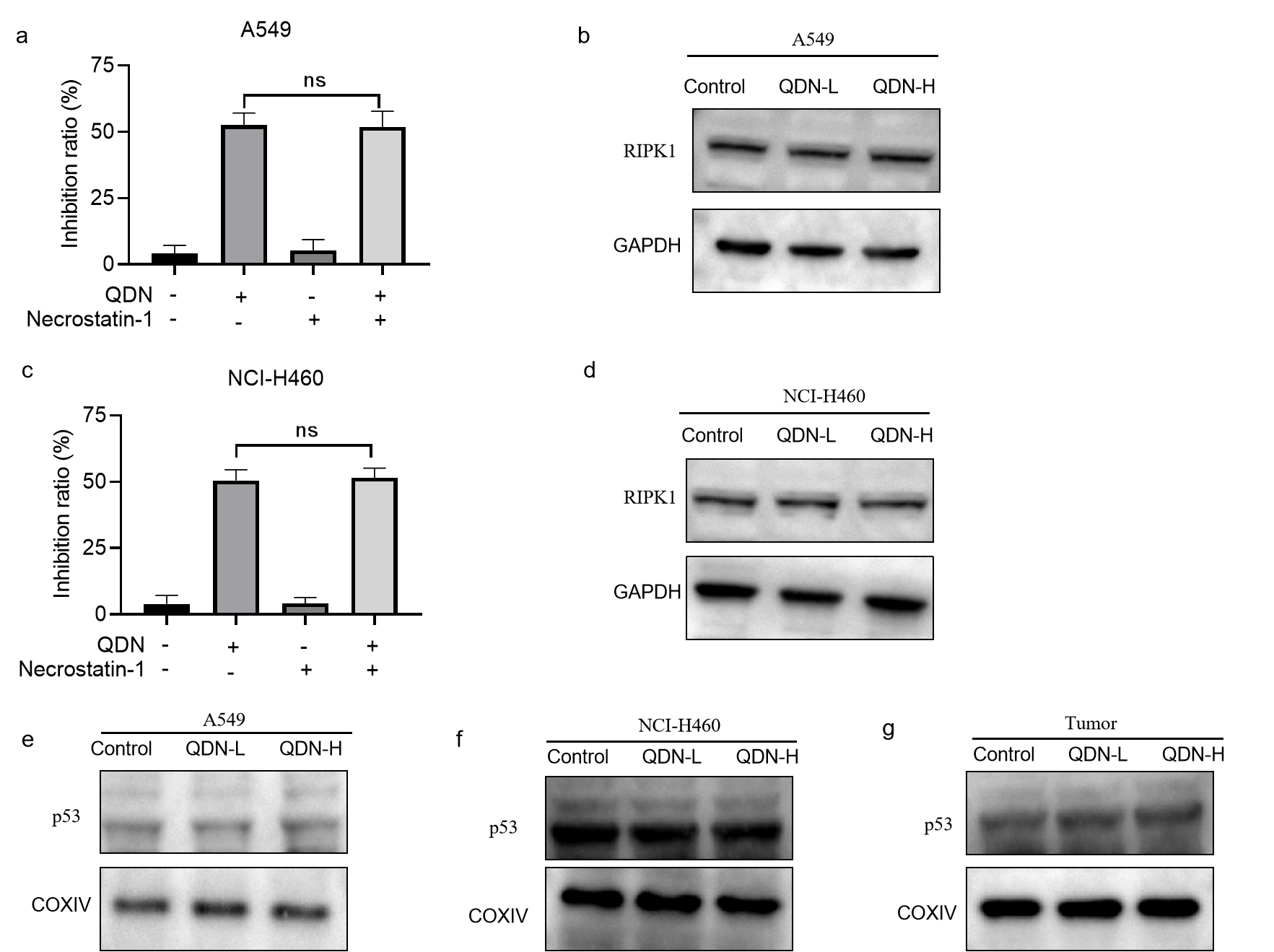


**Supplement figure 3**

#### MitoQ reduces ROS generation in QDN-treated lung cancer cells

**a** ROS production rates in A549 cells after QDN treatment for 48 h with or without MitoQ preconditioning. **b** ROS production rates in NCI-H460 cells after QDN treatment for 48 h with or without MitoQ preconditioning. Data are mean ± SD of duplicate experiments (n=3). Parametric variables were calculated using One-way ANOVA. **P* < 0.05, ***P* < 0.01, ****P* < 0.001. QDN-L: low dose of QDN, QDN-H: high dose of QDN.


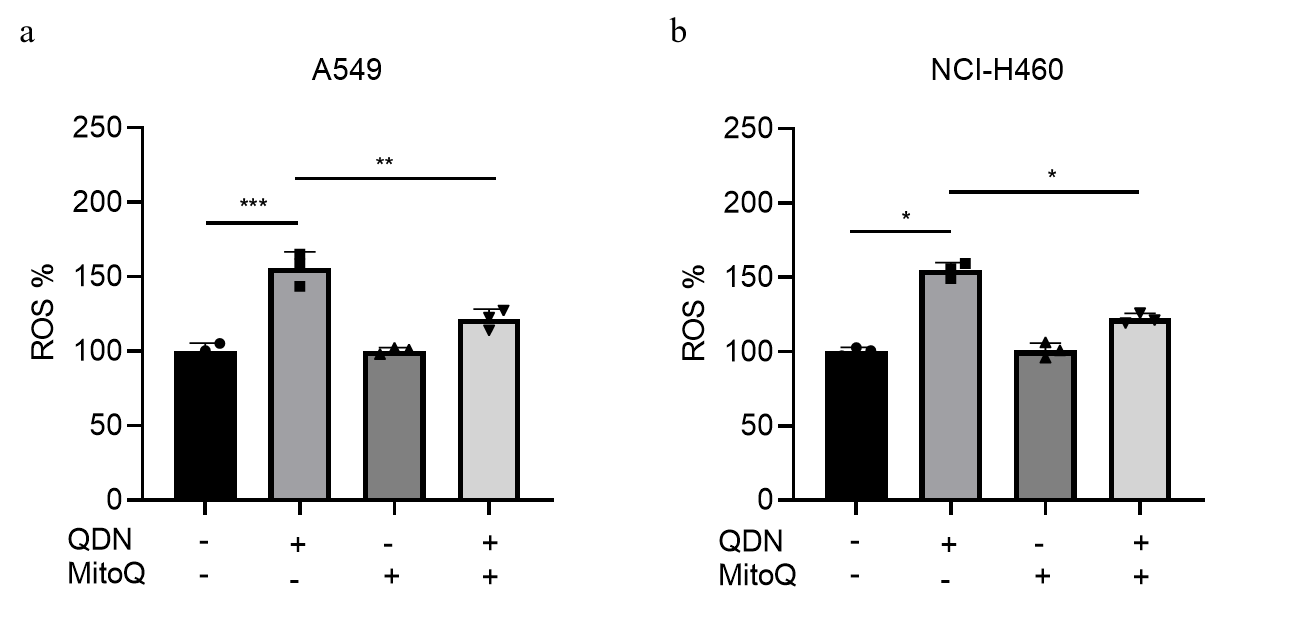

Supplement: Supplementary file 1 — Data S1. [file JCMM-28-e18353-s001.docx]
